# The β_1_-Subunit of Na_v_1.5 Cardiac Sodium Channel Is Required for a Dominant Negative Effect through α-α Interaction

**DOI:** 10.1371/journal.pone.0048690

**Published:** 2012-11-01

**Authors:** Aurélie Mercier, Romain Clément, Thomas Harnois, Nicolas Bourmeyster, Jean-François Faivre, Ian Findlay, Mohamed Chahine, Patrick Bois, Aurélien Chatelier

**Affiliations:** 1 Institut de Physiologie et Biologie Cellulaires, FRE 3511, CNRS/Université de Poitiers, Pôle Biologie Santé, Poitiers, France; 2 CHU de Poitiers, Poitiers, France; 3 Laboratoire de Physiologie des Cellules Cardiaques et Vasculaires, Institut de Physiologie et Biologie Cellulaires, FRE 3511 CNRS, Université François-Rabelais, Faculté des Sciences, Tours, France; 4 Institut universitaire en santé mentale de Québec, Université Laval, Québec, Québec, Canada; University of Waterloo, Canada

## Abstract

Brugada syndrome (BrS) is an inherited autosomal dominant cardiac channelopathy. Several mutations on the cardiac sodium channel Na_v_1.5 which are responsible for BrS lead to misfolded proteins that do not traffic properly to the plasma membrane. In order to mimic patient heterozygosity, a trafficking defective mutant, R1432G was co-expressed with Wild Type (WT) Na_v_1.5 channels in HEK293T cells. This mutant significantly decreased the membrane Na current density when it was co-transfected with the WT channel. This dominant negative effect did not result in altered biophysical properties of Na_v_1.5 channels. Luminometric experiments revealed that the expression of mutant proteins induced a significant reduction in membrane expression of WT channels. Interestingly, we have found that the auxiliary Na channel β_1_-subunit was essential for this dominant negative effect. Indeed, the absence of the β_1_-subunit prevented the decrease in WT sodium current density and surface proteins associated with the dominant negative effect. Co-immunoprecipitation experiments demonstrated a physical interaction between Na channel α-subunits. This interaction occurred only when the β_1_-subunit was present. Our findings reveal a new role for β_1_-subunits in cardiac voltage-gated sodium channels by promoting α-α subunit interaction which can lead to a dominant negative effect when one of the α-subunits shows a trafficking defective mutation.

## Introduction

Brugada syndrome (BrS) pathology is characterized by a unique electrocardiographic pattern of right bundle branch block with ST elevation in the right precordial which manifests as syncope or even sudden death caused by polymorphic ventricular tachycardia [1], [2]. This allelic disease is an autosomal dominant disorder caused by SCN5A mutations in 20% of patients [3], [4]. SCN5A encodes the α-subunit of the predominant cardiac sodium channel isoform known as Na_v_1.5. This α-subunit allows a fast inward flux of sodium ions through the plasma membrane of cardiomyocytes that underlies the initiation and propagation of cardiac action potentials. The α-subunit is associated with a β_1_-subunit which is known to play a critical role in the modulation of channel function and regulation of channel expression [5], [6]. As a consequence any functional defects of α or β_1_-subunits can result in abnormal cardiac conduction and excitability.

Many Na_v_1.5 loss-of-function mutations concern misfolded proteins that do not traffic properly to the plasma membrane [7]–[10]. This causes a decrease in Na_v_1.5 sodium channel surface expression and therefore reduces current density, which has been recognized as a common underlying mechanism of the BrS phenotype. Similarly, β_1_-subunit mutations that induce a marked effect on Na_v_1.5 sodium current density have been related to the BrS phenotype [11]. Since haploinsufficiency is proposed as a pathogenic mechanism underlying BrS, individuals heterozygous for trafficking defective SCN5A mutations should show a maximum loss of 50% of the Na_v_1.5 current. However, the incomplete penetrance of BrS questions the value of such an estimation.

One work on BrS reported a misfolded channel mutant whose surface expression was modulated by the co-expression of a Na_v_1.5 polymorphism [12]. In the same way, the L325R Na_v_1.5 mutant induces a reduction in WT Na_v_1.5 current [13]. An identical phenomenon has been observed for the voltage-gated calcium channel Ca_v_2.1 and was related to a dominant negative effect on Wild Type (WT) protein trafficking through an α-α subunit interaction [14], [15]. Na_v_1.5 and Ca_v_2.1 α-subunits share a common structure of four homologous domains of six transmembrane spanning segments associated to regulatory subunits. It is therefore possible that the mechanism of negative dominance characterized for Ca_v_2.1 also exists for Na_v_1.5. However, this possibility has been less investigated for Na_v_1.5 in spite of the fact that BrS is inherited as a dominant trait. In addition, whereas the β_1_-subunits influence the sodium current density [5], [6] its impact on a dominant negative effect has never been investigated.

This study had two objectives: (i) to assess the impact of the R1432G Na_v_1.5 trafficking defective mutant [7] on WT protein surface localization and function (ii) to investigate the potential implication of the regulatory β_1_-subunit.

## Materials and Methods

### DNA constructs

The cloning of the full length human Na_v_1.5/WT, Na_v_1.5/R1432G, Na_v_1.5/R1432G-FLAG and Na_v_1.5/WT-FLAG into pcDNA vectors was described previously [7]. The plasmid pEGFP-N2-Na_v_1.5 was a generous gift from Dr. Thomas Zimmer (Schiller Universität, Jena, Germany). The human sodium channel β_1_-subunit and CD8 were constructed in pIRES bicistronic vector (pCD8-IRES-β_1_).

### Cell culture and heterologous expression

HEK293T cells were maintained in high glucose DMEM (Dulbecco’s Modified Eagle’s Medium, Biowhittaker) containing 10% fetal bovine serum (Biowest) and 1% antibiotics (Gibco). Transient transfections were performed in HEK293T cells with pcDNA- Na_v_1.5 (WT, R1432G) and pCD8-IRES-β_1_ using a standard calcium phosphate precipitation method as previously described [16].

For co-transfection experiments, HEK293T cells were grown to 30% confluence in 100-mm culture dishes. For WT/WT condition, 3 µg plasmid of WT were co-transfected with 3 µg pCD8-IRES-β_1_. For heterozygous transfection conditions (WT/R1432G), 1.5 µg plasmid of WT and 1.5 µg plasmid of mutant Na_v_1.5 were co-transfected with 3 µg pCD8-IRES-β_1_ or 3 µg pCD8-IRES. For WT/(-) and (-)/R1432G conditions, 1.5 µg empty vector was added to 1.5 µg of WT or mutant plasmids and to 3 µg pCD8-IRES-β_1_, so that all transfection conditions contain the same amount of DNA.

All electrophysiological and biochemical experiments were carried out forty-eight hours after transfection.

### Co-immunoprecipitation and immunoblotting

Forty-eight hours post-transfection, the cells were washed with ice-cold PBS (Phosphate Buffered Saline: 137 mM NaCl, 1.5 mM KH_2_PO_4_, 2.7 mM KCl and 8 mM Na_2_HPO_4_, pH 7.4). Plates were scraped in lysis buffer (50 mM Tris-HCl, 150 mM NaCl, 5 mM EDTA, 0.05% NP-40, 1% deoxycholic acid, 1% Triton X-100, 0.1% SDS) containing protease inhibitors (Protease Inhibitor Cocktail, Sigma) and phosphatase inhibitor cocktail (PhoSTOP, Roche). Lysates were then incubated for 30 min on ice and clarified at 1750 g for 5 min at 4°C. Protein contents were measured using DC protein assay (Biorad) with BSA as a reference.

For co-immunoprecipitation experiments, 500 µg of supernatant were mixed into 500 µl of NET buffer (50 mM Tris-HCl pH 7.4, 150 mM NaCl, 5 mM EDTA, 0.05% NP-40, pH 7.4). Incubation was conducted overnight at 4°C with 2 µg of anti-FLAG M2 antibodies (Sigma) with continuous agitation. To precipitate the immune complexes, samples were incubated with 30 µl of protein G-sepharose (100 mg/mL) for 1 h at 4°C. Bead-bound complexes were washed three times with NET buffer, eluted at 37°C for 1 h in the Laemmli sample buffer and analyzed by immunoblotting. As negative and positive controls, 500 µg of transfected cells expressing the β_1_-subunit and both tagged channels were immunoprecipitated with non-specific mouse IgG and anti-FLAG antibodies.

Total cell lysates and immunoprecipitated proteins were separated by SDS-PAGE using 6% polyacrylamide gels. Proteins were transferred and nitrocellulose membrane were then probed with primary antibodies rabbit polyclonal SP19 anti-pan-Na_v_ (1∶1000, Alomone Labs) or rabbit anti-GFP (1∶1000, Invitrogen). Anti-rabbit horseradish peroxidase-conjugated secondary antibodies (1∶5000, Interchim) were used before revealing membrane with ECL chemiluminescent substrate (GE Healthcare). Signal intensities of bands in the immunoblots were quantified using Scion image analysis software (Scion Corp).

### Immunofluorescence staining

Twenty-four hours after transfection, cells were trypsinized and transferred on poly-D-lysine coated glass coverslips.

HEK293T cells were washed and fixed with 3% paraformaldehyde in PBS for 10 min. Non-specific binding sites were blocked with PBS containing 5% BSA (blocking buffer) for 30 min. Primary mouse anti-FLAG M2 antibodies (Sigma, 1∶1000) diluted in blocking buffer were added to the cells 1 h at 4°C. Cells were then washed with PBS, permeabilized with 0.1% Triton X-100 for 20 min and incubated overnight with primary rabbit anti-Na_v_1.5 antibodies (Alomone Labs, 1∶200). After PBS washes, appropriate fluorophore-conjugated secondary antibodies diluted in blocking buffer were added to the cells 1 h at room temperature. Coverslips were rinsed and mounted on slides in Mowiol (Sigma). Images were acquired and processed by using an inverted confocal laser-scanning microscope (FV1000, Olympus) and with FV10-ASW software (Olympus). For each experiment, acquisition settings were kept constant between comparative conditions.

Secondary antibodies used were Alexa Fluor 555-conjugated chicken anti-mouse IgG (1∶500) and Alexa Fluor 488-conjugated donkey anti-rabbit IgG (1∶1000) from Molecular Probes (Invitrogen). The specificity of secondary antibodies was confirmed by the absence of a signal in transfected and non-transfected cells when the primary antibody was omitted.

### Luminometric surface expression measurements

HEK293T cells were transfected with Na_v_1.5/WT-FLAG (with or without Na_v_1.5/R1432G and β_1_). Twenty-four hours later, cells were trypsinized and reseeded at 4×10^5^ cells per well into fibronectin-coated 24-well Visiplate (Perkin Elmer).

Initial procedure for luminometric experiments was similar to fixation and saturation steps described for immunocytochemistry with an optional permeabilization treatment. After fixation, half of transfected cells were permeabilized with PBS 1% BSA and 0.1% Triton X-100 for 20 min at room temperature. For each condition, half of the wells were then labelled with anti-FLAG antibody diluted at 1∶1000 in blocking buffer for 1 h at 4°C. After PBS washes, all cells were incubated for 30 min with anti-mouse antibody coupled to horseradish peroxidase (1∶3000, Interchim). Cells were then washed with PBS before addition of freshly prepared SuperSignal ELISA Femto Maximum Sensitivity Substrate (Pierce). The plates were immediately read in a luminometer (Mithras LB 940, Berthold Technologies) and the luminescence (expressed as relative light units, RLU) was integrated over 0.1 s. To minimize background luminescence level due to non-specificity of secondary antibody, signals detected in cells labelled with both primary and secondary antibodies, were subtracted from those measured from wells in which primary antibody was omitted.

### Patch clamp recordings

For patch clamp experiments, twenty-four hours after transfection, cells were trypsinized from the 100-mm dish and redistributed on 35-mm dishes at a density of 4×10^4^ cells per dish in DMEM. The co-transfection of selected Na_v_1.5 constructs with pCD8-IRES-β_1_ or pCD8-IRES allowed the identification of efficiently transfected cells for electrophysiological recordings. Membrane CD8 antigen expression was visualized by using anti-CD8 coated beads (Dynabeads CD8, Invitrogen, Dynal).

Macroscopic sodium currents from transfected cells were recorded using the whole-cell configuration of the patch clamp technique. All cells binding CD8 beads expressed sodium currents (I_Na_) and were considered as efficiently transfected. For I_Na_ recordings, low resistance electrodes (1.5–2 MΩ) were drawn from borosilicate glass capillaries (Harvard apparatus) and were coated with the silicone elastomer HIPEC® R6101 (Dow-Corning, Midland, MI, USA) to minimize their capacitance. The liquid junction potential of 4 mV was corrected prior to experiments. Intracellular solution contained 35 mM NaCl, 105 mM CsF, 10 mM EGTA and 10 mM HEPES, pH adjusted to 7.4 with CsOH. Bath solution was made with 60 mM NaCl, 2 mM KCl, 1.5 mM CaCl_2_, 1 mM MgCl_2_, 10 mM glucose, 10 mM HEPES and 90 mM CsCl, pH adjusted to 7.4 with NaOH.

All electrophysiological experiments were performed at room temperature (22–24°C). To ensure that current amplitudes were stabilized before recording begin measurements were carried out 5 min after obtaining whole-cell configuration. Experiments were performed using the same sequence of protocols between cells.

Currents were recorded with an Axopatch 200A amplifier (Axon Instruments) and voltage-clamp command pulses were controlled and data acquired using pClamp10.2 software (Molecular Devices). Membrane currents were filtered at 5 kHz and were sampled at a rate of 100 kHz for analysis and capacitive transients were cancelled and voltage errors were minimized with 80% series resistance compensation. Linear leak currents were removed using P/4 leak subtraction.

For current-voltage relationships, cells were held at -140 mV and stepped in 10-mV increments from -100 to 40 mV for 50 ms. To obtain I-V curves, peak currents divided by cell membrane capacitance were plotted as a function of membrane potential. Cell membrane capacitance was obtained as telegraph readout of cell capacitance compensation. The voltage dependence of activation was determined from the relative membrane conductance as a function of potential using the formula G_Na_ = I_Na_/(V_m_–V_rev_), where G_Na_ is peak conductance and I_Na_ is peak sodium current for the test potential V_m_. V_rev_ is the estimated reversal potential of the sodium current determined for each cell individually. The resulting sodium channel conductance was normalized to the maximum response for each cell. Activation curves were fitted with Boltzmann function: G/G_max_  = 1/(1+exp [(V-V_1/2_)/*k*]), where G_max_ represents the maximum conductance and V_1/2_ and *k* represent the half-maximum voltage of activation and the Boltzmann constant, respectively.

The voltage-dependence of steady-state inactivation was estimated by measuring the peak I_Na_ during a 20-ms test pulse to −30 mV, which followed a series of 500-ms prepulses of membrane potentials between −140 and 0 mV from a holding potential of −140 mV. Peak inward currents were normalized to the maximum peak current I_max_ of each cell. The inactivation data were also fitted with a Boltzmann equation.

### Data analysis and statistics

Data were analyzed using Clampfit 10.2 (Molecular Devices), Origin 8.0 (Microcal Software), and Microsoft Excel. Statistical analyses were performed with Student *t*-test or one-way analysis of variance (ANOVA) with Bonferroni's post hoc tests for multiple comparisons using Origin 8.0 (Microcal Software, Inc.). A value of P<0.05 was considered statistically significant. All values are reported as mean ± SEM.

## Results

### Sodium current properties of WT Na_v_1.5 co-expressed with the R1432G mutant

**Figure 1:**
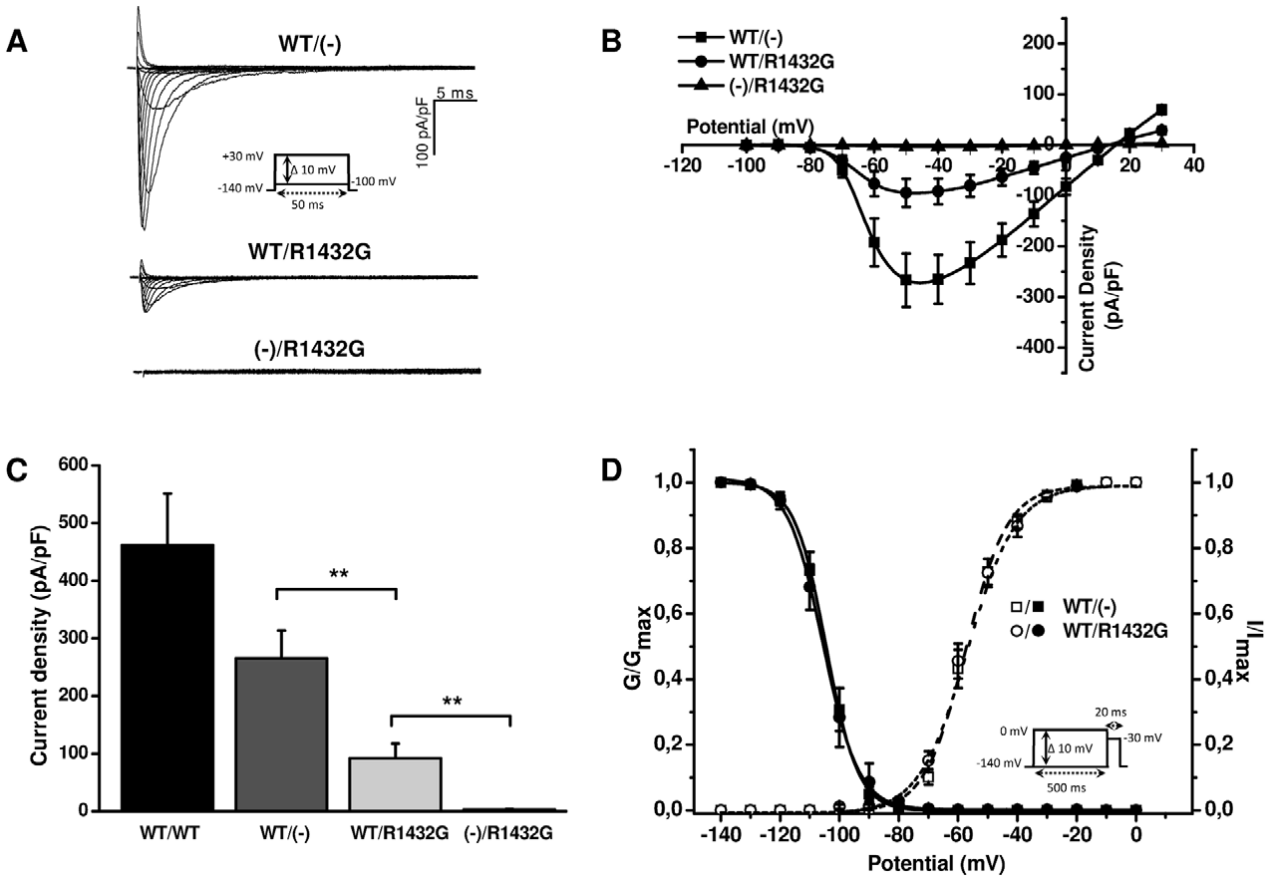
Functional impact of R1432G mutant co-expression upon WT sodium channels in the presence of β_1_-subunit. **A.** Representative whole-cell sodium current recordings from HEK293T cells expressing WT and/or R1432G Na_v_1.5 channels and the β_1_ auxiliary subunit. Current traces are presented as membrane current density. The inset shows the voltage-clamp protocol. **B.** Current-voltage relationships of Na_v_1.5 currents measured from 7–11 cells. **C.** Average peak current densities of WT and/or R1432G channels at -40 mV. **, P<0.01 *versus* WT/R1432G (Student *t*-test). **D.** Voltage-dependence of activation (open symbols, n = 11) and inactivation (filled symbols, n = 7–9) of Na_v_1.5 channels. Inactivation and activation data were measured with standard pulse protocols shown in inset and in Figure 1A respectively. Activation and inactivation curves were generated and fitted with a Boltzmann equation to obtain biophysical parameters summarized in Table 1.

Figures 1A and 1B illustrate representative cell current recordings and current-voltage relationships obtained from HEK293T cells transfected with WT (WT/(-)), WT and R1432G (WT/R1432G) or R1432G Na_v_1.5 channel cDNA ((-)/R1432G). Each transfection included cDNA for the β_1_ subunit. The WT Na_v_1.5 channel showed a robust voltage-gated Na current (n = 11) which was activated by voltage steps to ∼−80 mV from the holding potential of -140 mV and reached a peak current amplitude with voltage-steps to between −50 and −40 mV. Cells transfected with the R1432G Na_v_1.5 channel showed no visible voltage-gated Na current (n = 7). To represent heterozygous Brugada patients, co-expression of equal amounts of WT and R1432G Na_v_1.5 channel cDNA led to clearly less functional Na current (n = 11) than WT alone.

**Table 1:** Gating parameters of WT and R1432G Na_v_1.5 channels expressed in HEK293T cells.

	*Activation*			*Inactivation*		
Expressed α/β subunits	V_1/2_ (mV)	*k* (mV)	*n*	V_1/2_ (mV)	*k* (mV)	*n*
WT/(-) + β_1_	−57.6±1.5	6.29±0.37	*11*	−104.5±1.6	−4.85±0.14	*7*
WT/R1432G + β_1_	−57.6±1.9	7.33±0.45	*11*	−105.5±2.6	−4.11±0.48	*9*
WT/(-) – β_1_	−58.0±1.6	7.52±0.51	*9*	−113.1±2.0^*^	−5.33±0.20^††^	*7*
WT/R1432G – β_1_	−59.8±1.1	7.12±0.26	*10*	−114.9±1.0^**,†^	−5.26±0.17†	*7*

Abbreviations are: *n*, number of cells per group; *k*, slope factor of voltage dependence of (in)activation (mV) and V_1/2_, voltage of half-maximal (in)activation (mV). Statistically significant results were determined using one-way analysis of variance (ANOVA) with Bonferroni's post hoc tests. ^*^, P<0.05; ^**^, P<0.01 (*vs* WT/(-) + β_1_); ^†^, P<0.05; ^††^, P<0.01 (*vs* WT/R1432G + β_1_).

To test whether this reduction of WT current due to the inclusion of R1432G cDNA resulted from saturation of transcription or translation machinery we doubled the amount of WT cDNA. Figure 1C shows that this increased peak Na current to 461.7±89.4 pA/pF (WT/WT, n = 5) compared with 265.4±48.2 pA/pF (n = 11) for half the amount of WT cDNA (WT/(-); P = 0.05304, *t*-test). The addition of R1432G (WT/R1432G, n = 11) resulted in a significant decrease of Na current by 65% to 91.7±25.4 pA/pF (P<0.01, *t*-test).

These results show that first, the protein expression systems of the cells were not saturated by the transfection of WT and R1432G DNA and secondly, that the transfection of R1432G had a strong dominant negative effect upon the current density of WT Na_v_1.5.

Activation and inactivation parameters were measured in cells expressing the different Na_v_1.5 proteins to examine whether this dominant negative effect was associated with changes in the biophysical properties of the Na_v_ channel. Activation and inactivation parameters were not significantly different in WT/R1432G compared to WT/(-) (Figure 1D, Table 1). Recovery from inactivation was also unaffected by co-expression of the mutant R1432G with WT (data not shown).

### The R1432G mutant reduces localization of Na_v_1.5 WT at the cell surface

Our aim was to characterize this dominant negative effect by distinguishing expression and localization of WT from mutant Na_v_1.5 channels. As above, all cells were also transfected with the β_1_-subunit.

One hypothesis is that R1432G mutants would target WT channels to the proteasome by the endoplasmic reticulum quality control. However, for each transfection condition, treatment with the proteasome inhibitor MG132 (10 µM, 6 h) revealed a similar degradation level of WT and mutant Na_v_ channels (Figure S1). On the other hand, this experiment confirmed that the amounts of Na_v_1.5 WT and R1432G proteins were similar in the different transfection conditions.

To further characterize this dominant negative effect we used a FLAG-tagged construct in which an extracellular FLAG epitope was introduced into the WT channel between transmembrane segments S5 and S6 of domain I. This allowed us to distinguish between the amount and localization of WT and mutant Na_v_1.5 channels.

**Figure 2:**
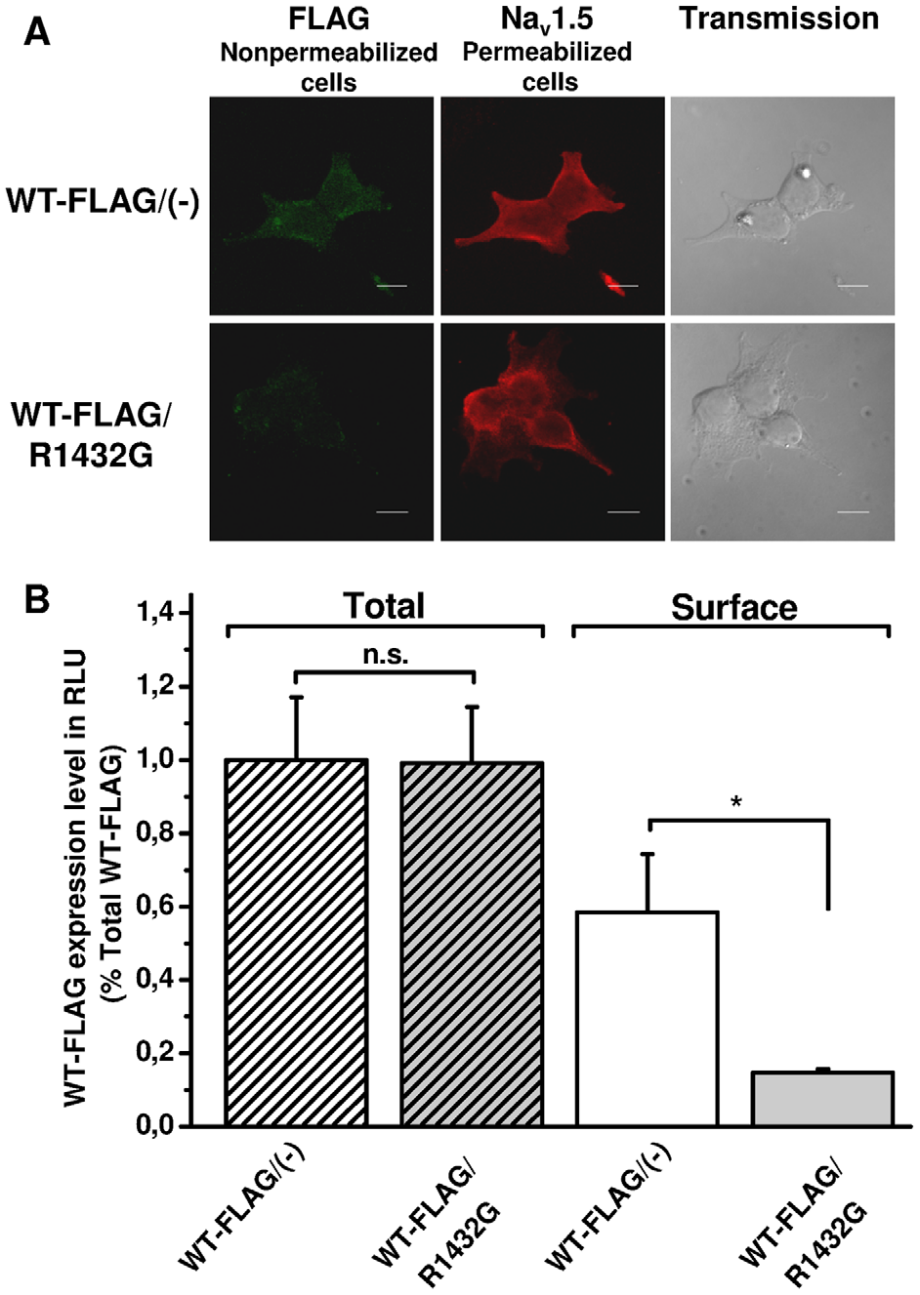
R1432G mutant alters WT channel localization to the cell membrane in the presence of β_1_-subunit. **A.** Confocal imaging of Na channels in cells co-expressing R1432G mutant and Na_v_1.5/WT-FLAG or expressing Na_v_1.5/WT-FLAG alone. Immunocytochemistry with anti-FLAG antibodies was performed first on fixed non-permeabilized cells to detect surface FLAG-tagged WT channels (green, left panels). The same cells were then permeabilized to label total mutant and WT Na_v_1.5 proteins with anti-Na_v_1.5 antibodies (red, middle panels). Light transmission imaging of the HEK293T cells are presented on the right panels. Scale bar, 10 µm. **B.** Quantification by luminometry of FLAG-tagged Na_v_1.5/WT channels in the presence (gray bars) or the absence (white bars) of R1432G subunits in HEK293T cells. Surface and total amount of WT-FLAG channels was measured from non-permeabilized (solid bars) and permeabilized (hatched bars) cells respectively. Data are presented as relative light unit (RLU) normalized to the total WT-FLAG/(-) condition. Data are expressed as mean ± SEM of six independent experiments. *, P<0.05, indicates significant difference with surface WT-FLAG/( ) condition (*t*-test). All experiments were realized in the presence of the β_1_-subunit.

Confocal microscopy with double immunofluorescence staining was performed first with anti-FLAG antibodies under non-permeabilized conditions followed by a second labelling step with anti-Na_v_1.5 antibodies on the same cells after permeabilization (Figure 2A). This enabled us to estimate in the same cells the surface localization of FLAG-tagged WT Na_v_1.5 proteins as well as the total expression of mutant and WT channels. HEK293T cells expressing the WT-FLAG channels alone or with R1432G mutants exhibited a similar total Na_v_1.5 staining (Figure 2A, middle panels). In contrast, co-expressing WT and R1432G channels reduced plasma membrane localization of WT-FLAG channels (Figure 2A, left panels).

This observation was supported by luminometric assays which quantified total and surface Na_v_1.5 WT expression in the presence or in the absence of the R1432G mutant (Figure 2B). Total and membrane expressions were measured on permeabilized and non-permeabilized cells, respectively. The presence of the R1432G mutant did not affect the total amount of WT channels (respectively 100±17.2% to 99.2±15.3%). In contrast, R1432G reduced surface expression of WT channels to 14.6±1.0% compared with cells expressing WT channels alone (58.5±15.9%, n = 6, P<0.05, *t*-test). This 75% decrease in membrane protein amount is similar to the current reduction observed in patch clamp experiments (Figure 1C).

### The dominant negative effect of R1432G Na_v_1.5 α-subunits upon WT Na current requires the presence of β_1_-subunits

**Figure 3:**
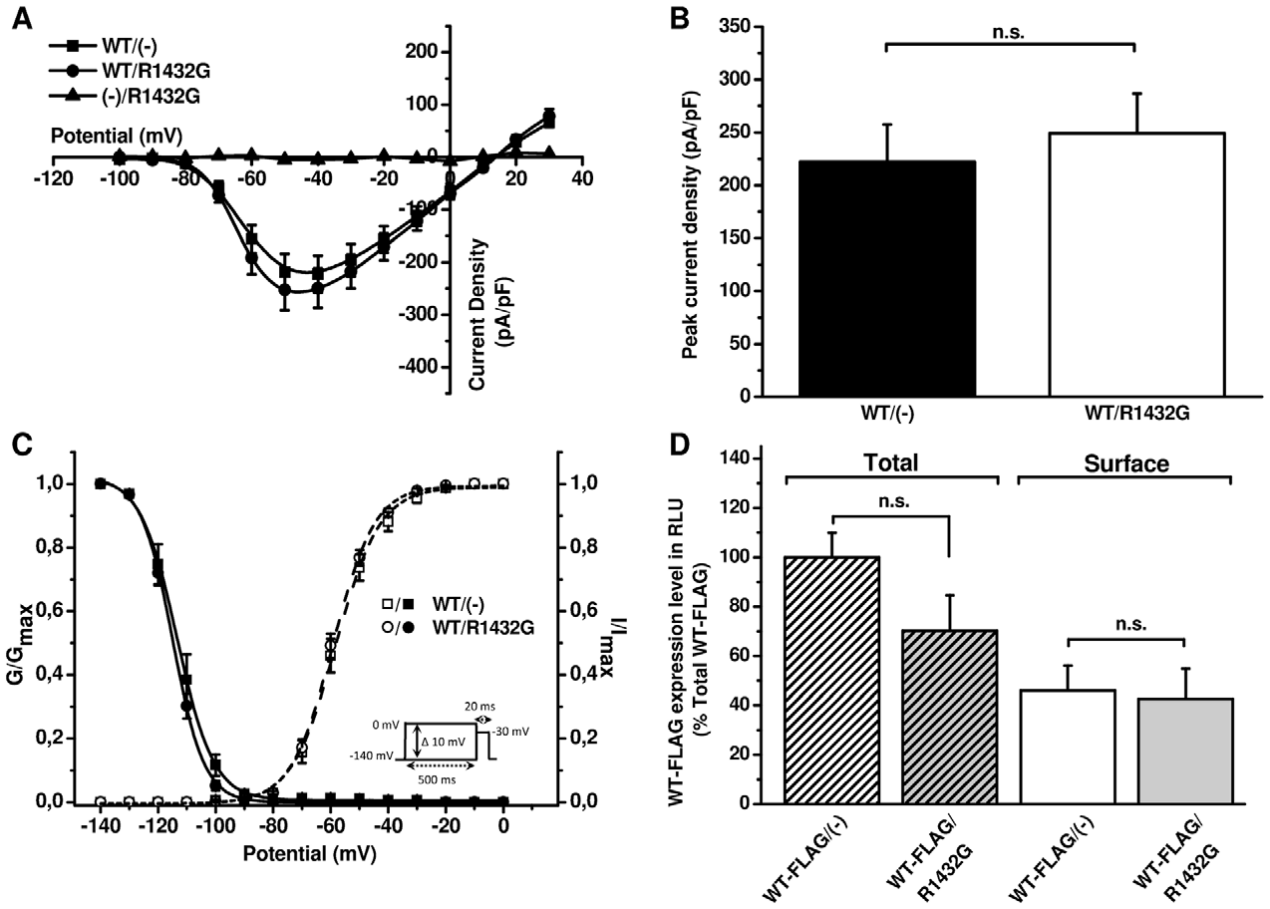
Abolition of R1432G dominant-negative effect in the absence of the β_1_-subunit. **A.** IV curves of Na_v_1.5 currents recorded from HEK293T cells transfected with WT and/or R1432G channels without β_1_-subunit (n = 9–10). **B.** Average peak current densities WT and/or R1432G channels at -40 mV. n.s., p>0.05 *versus* WT/R1432G condition (Student *t*-test). **C.** Voltage-dependence of activation (open symbols, n = 9–10) and inactivation (filled symbols, n = 7) of WT and/or R1432G Na_v_1.5 expressed in HEK293T without β_1_. Inactivation and activation data were obtained using standard pulse protocols shown in inset and in Figure 1A respectively. (In)activation parameters are summarized in Table 1. **D.** Quantification by luminometric ELISA assay of FLAG-tagged Na_v_1.5/WT channels alone (white bars, n = 6) or with R1432G (grey bars, n = 6) in the absence of β_1_-subunit. The bar chart shows surface and total amount of WT-FLAG channels from nonpermeabilized cells (solid bars) and permeabilized cells (hatched bars) respectively in the absence of β_1_-subunit. Data are presented as relative light unit (RLU) normalized to the total WT-FLAG/(-) condition and are expressed as mean ± SEM. n.s. indicates no significant difference with surface WT-FLAG/(-) condition (*t*-test).

We tested the possible implication of the regulatory β_1_-subunit on the dominant negative effect of R1432G upon WT α-subunit surface expression and function. In the absence of β_1_-subunits whole-cell patch clamp current recordings from cells expressing WT or co-expressing WT/R1432G produced the same membrane current densities (Figure 3). In both conditions, sodium currents exhibited robust current voltage relationships similar to those obtained with WT in presence of β_1_-subunit (Figures 1B and 3A). In the absence of β_1_-subunits when R1432G was co-expressed with the WT, the peak Na current density was not significantly different from that of WT expressed alone (222.4±35.0 pA/pF, n = 9, for the WT(-) *versus* 249.3±37.24 pA/pF for WT/R1432G, P>0.05, n = 10, Figure 3B). As expected, the mutant R1432G expressed alone in the absence of β_1_-subunits ((-)/R1432G, n = 3) also did not produce any membrane inward current.

Activation and inactivation parameters were not significantly different between WT/R1432G and WT/(-) in the absence of β_1_-subunits (Figure 3C, Table 1) though the voltage-dependence of Na_v_ current inactivation was significantly shifted to more negative voltages by comparison with the α-subunits expressed with β_1_-subunits (Table 1).

Luminometric assays were then carried out in the absence of β_1_ (Figure 3D) where total amount of FLAG-tagged WT channels was not significantly different by comparison with WT/R1432G (100±9.9% *versus* 70.1±14.5% (n = 6, p>0.05, *t*-test)). In contrast to cells also expressing the β_1_-subunit (Figure 2), in the absence of auxiliary subunits the co-expression of R1432G mutant did not alter the surface expression of WT channels (respectively 45.9±10.2% and 42.6±12.3%, n = 6, p>0.05, *t*-test).

Thus, in the absence of the β_1_-subunit there is no dominant negative effect of R1432G on cell surface localization or function of WT channels.

### Interaction between R1432G and WT Na_v_1.5 α-subunits depends on β_1_-subunit expression

**Figure 4:**
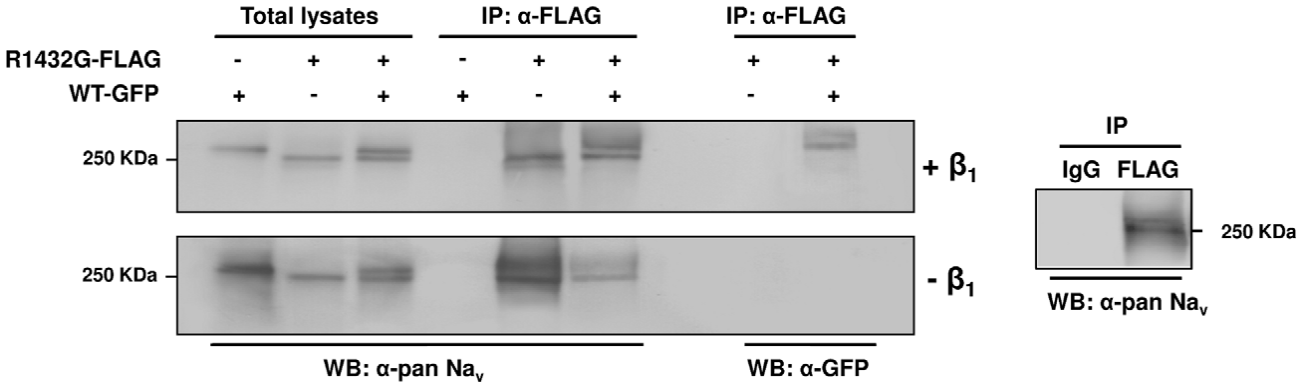
Na_v_1.5 α-α subunits interaction is dependent on the auxiliary β_1_-subunit. HEK293T cells were transfected with FLAG-tagged R1432G and/or GFP-tagged WT channels in the absence (bottom panel) or the presence (top panel) of β_1_-subunit. Immunoprecipitations were performed with anti-FLAG M2 antibodies. Equal amounts of total lysates (15 µg) and immunoprecipitated proteins were immunoblotted with anti-pan-Na_v_ and anti-GFP antibodies to respectively detect both tagged forms or GFP-WT channels. Negative and positive controls are shown in the right panel. (WB: Western Blot; IP: immunoprecipitation).

Finally, we investigated the interaction between mutant and WT Na_v_1.5 α-subunits in the absence and the presence of β_1_-subunits. Two distinct vectors encoding FLAG-tagged R1432G mutant and GFP-tagged WT α-subunits were co-transfected in HEK293T cells. The fusion of the GFP-tag to Na_v_1.5 α-subunits induces an approximately 30-KDa upward mobility shift of Na_v_ signals compared with those of FLAG-tagged channels in co-immunoprecipitation of total lysates (Figure 4).

We took advantage of this difference to discriminate between the different tagged proteins using the same anti-Na_v_ antibody. Total lysates indicates that both FLAG-tagged R1432G mutant and GFP-tagged WT have similar expression level when they are co-expressed. Co-immunoprecipitation experiments, which were carried out with anti-FLAG antibodies on tagged R1432G mutants, demonstrate that GFP-WT α-subunits strongly interact with mutant channels in the presence of β_1_-subunits (Figure 4, top panel). When co-immunoprecipitations were performed without β_1_-subunit, no WT Na_v_1.5 signal was detectable in immunoblots with anti-GFP which indicates an absence of α-subunit interaction (Figure 4, bottom panel). Surprinsingly, without β_1_-subunit, a signal was detected with anti-pan-Na_v_, at higher molecular weight when FLAG-tagged R1432G was expressed. Concentration of the tagged R1432G mutants by immunoprecipitation could highlight another form of the protein that was absent in the presence of the β_1_-subunit (Figure 4, Top panel). Similar experiments performed between differently tagged WT α-subunits revealed that α-subunits interact with each other by a β_1_-subunit dependent mechanism (Figure S2).

## Discussion

This study clearly demonstrates for the first time that the β_1_-subunit is responsible for physical interaction between Na_v_1.5 α-subunits. We show that when a Na_v_1.5 trafficking defective mutant and WT are co-expressed, this interaction leads to a strong dominant negative effect and reduces WT Na_v_1.5 surface localization. These results reveal a new role for voltage-gated sodium channel β-subunits.

R1432G Na_v_1.5 is a misfolded α-subunit mutant which can contribute to BrS [7], [17]. By itself the expression of R1432G results in no inward Na current because it is retained in intracellular compartments rather than being trafficked to the cell membrane. Here we show that this mutant α-subunit can have a strong negative dominance on the WT membrane Na current. This effect is not associated with any modification of functional sodium current gating properties. Dominant negative effects of mutant channels on WT are poorly understood for voltage-gated sodium channels. As these channels are not known to form multimeric complexes the co-expression of WT and mutant subunits have not been systematically realized. To date, only two studies support such an alteration in sodium membrane currents. However the mechanisms by which those mutants interfere with WT channels have not been elucidated yet [13], [18].

A dominant negative effect has been described for α-subunits of neuronal calcium channels Ca_v_2.1 where misfolded α-subunits led to increased destruction of WT through the endoplasmic reticulum-associated degradation pathway [14]. This was not the case in our investigation of Na_v_1.5 where the level of protein degradation blocked by MG132, an inhibitor of the proteasome degradation pathway [19], was similar in all our expression conditions (Figure S1). As a consequence, an enhanced degradation of WT Na_v_1.5 cannot be responsible for the dominant negative effect.

The FLAG-tagged Na_v_1.5 in luminometric experiments show that the dominant negative effect occurs through a reduction in WT Na_v_1.5 α-subunits in the plasma membrane without changing the total amount of protein. The most probable underlying mechanism is an interaction between mutant and WT α-subunits that would prevent WT α-subunit transport to the cell surface. The co-immunoprecipitation experiments do demonstrate a physical interaction between α-subunits which concords with a previous suggestion of interaction between Na_v_1.5 fragments with FRET [12].

The fully functional cardiac muscle voltage-gated sodium channel (Na_v_1.5) is a monomeric structure composed of a single α pore-forming subunit and the auxiliary β_1_-subunit [20]. Although the α-subunit is capable of cell surface expression and functional voltage-gated Na current, the β_1_-subunit contributes to and improves intracellular trafficking, increases cell membrane insertion and modulates the kinetics of channel behavior [6], [21]. The major novelty of our study is the observation that β_1_-subunits were required for the negative dominant interaction between R1432G and WT α-subunits. β_1_-subunits are known to interact homophilically through their extracellular cell adhesion molecule domain [22]. Such an interaction could form the basis of a multimeric complex of two α and two β_1_-subunits that would be responsible for the dominant negative effect of the R1432G mutant upon WT α-subunits by retention in the intracellular environment.

Our findings reveal a new role for β_1_-subunits in supporting interaction between α-subunits of the voltage-gated sodium channel. As a consequence the impact of β_1_-subunit mutations on this interaction offers new possibilities in pathologies such as BrS, epilepsia or febril seizures [5], [11]. However, whether this α/β multimeric complex exists at the plasma membrane or only at the intracellular level still has to be elucidated.

## Supporting Information

Figure S1 **R1432G and WT co-expression effect on proteasome-mediated degradation of Na_v_1.5 channels.** Western blots were performed on total lysates from HEK293T cells expressing either WT channels alone (WT/WT), WT channels with empty vector (WT/(-)), WT and mutant channels (WT/R1432G) or mutant channels with empty vector ((-)/R1432G). **A.** Representative blots of three independent experiments are shown. Cells were treated with 10 µM MG132 or vehicle DMSO for 6 hours prior to cell lysis. β-actin was used as loading control. Proteins were probed with primary antibodies rabbit polyclonal SP19 anti-pan-Na_v_ (1∶1000, Alomone Labs) and mouse monoclonal anti-β-actin (1∶10000, Sigma). **B.** Quantification of proteasomal degradation. Na_v_1.5 signal was quantified by densitometric analysis and was first normalized to β-actin band intensity. For each experiment, Na_v_1.5 expression levels are expressed as percentage of DMSO control condition. No significant difference was reported between all MG132 conditions (n.s., ANOVA).
(TIF)

Figure S2 **The auxiliary β_1_-subunit is responsible for the association of WT Na_v_1.5 α-α subunits.** HEK293T cells were transfected with FLAG-tagged and/or GFP-tagged WT channels in the presence (left panels) or the absence (right panels) of the β_1_-subunit. Immunoprecipitations were performed with anti-FLAG M2. Equal amounts of total lysates (15 µg) and immunoprecipitated proteins were immunoblotted with anti-pan-Na_v_. (WB: Western Blot; IP: immunoprecipitation).
(TIF)
