# Supplementary figures and images for: The β1-Subunit of Nav1.5 Cardiac Sodium Channel Is Required for a Dominant Negative Effect through α-α Interaction
Source: PLoS One. 2012 Nov 1;7(11):e48690. doi: 10.1371/journal.pone.0048690 (PMC3486797; doi:10.1371/journal.pone.0048690)

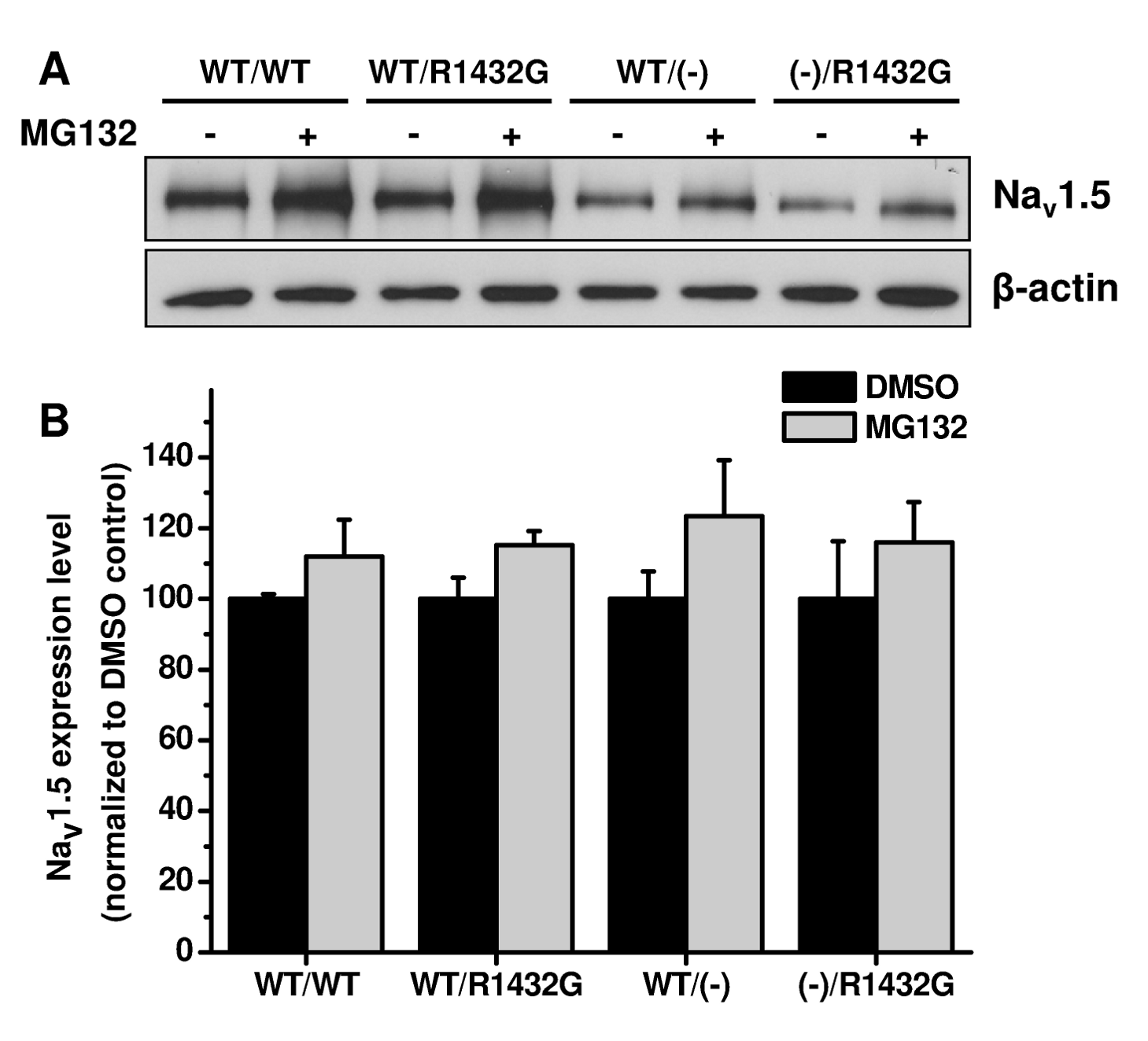

Supplement: Figure S1 — R1432G and WT co-expression effect on proteasome-mediated degradation of Nav1.5 channels. Western blots were performed on total lysates from HEK293T cells expressing either WT channels alone (WT/WT), WT channels with empty vector (WT/(-)), WT and mutant channels (WT/R1432G) or mutant channels with empty vector ((-)/R1432G). A. Representative blots of three independent experiments are shown. Cells were treated with 10 µM MG132 or vehicle DMSO for 6 hours prior to cell lysis. β-actin was used as loading control. Proteins were probed with primary antibodies rabbit polyclonal SP19 anti-pan-Nav (1∶1000, Alomone Labs) and mouse monoclonal anti-β-actin (1∶10000, Sigma). B. Quantification of proteasomal degradation. Nav1.5 signal was quantified by densitometric analysis and was first normalized to β-actin band intensity. For each experiment, Nav1.5 expression levels are expressed as percentage of DMSO control condition. No significant difference was reported between all MG132 conditions (n.s., ANOVA). (TIF) [file pone.0048690.s001.tif]

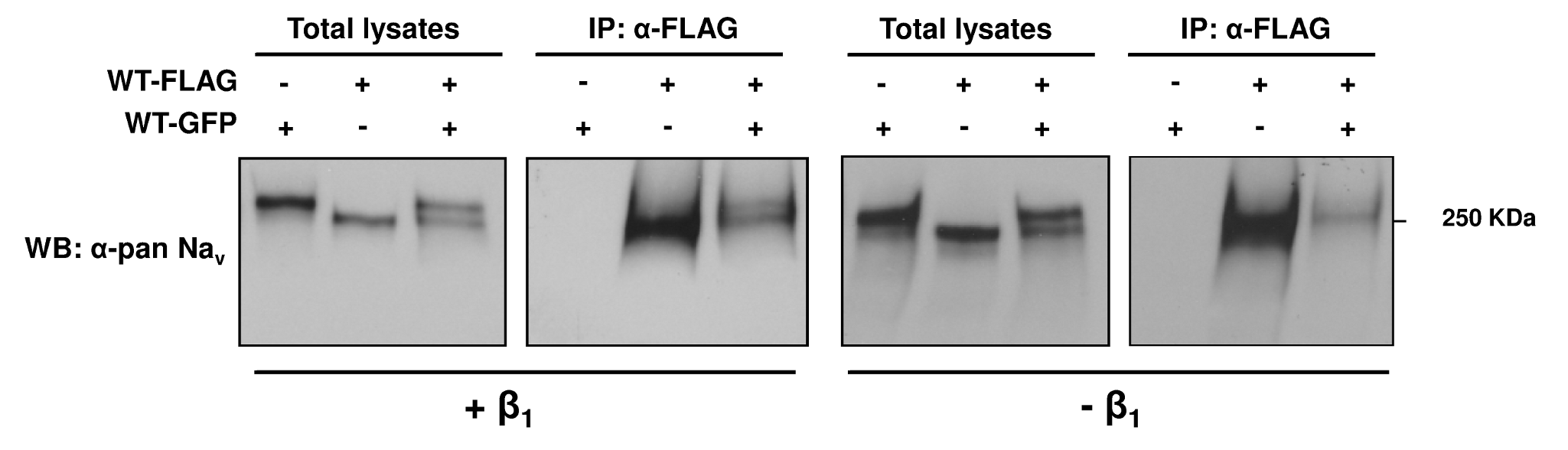

Supplement: Figure S2 — The auxiliary β1-subunit is responsible for the association of WT Nav1.5 α-α subunits. HEK293T cells were transfected with FLAG-tagged and/or GFP-tagged WT channels in the presence (left panels) or the absence (right panels) of the β1-subunit. Immunoprecipitations were performed with anti-FLAG M2. Equal amounts of total lysates (15 µg) and immunoprecipitated proteins were immunoblotted with anti-pan-Nav. (WB: Western Blot; IP: immunoprecipitation). (TIF) [file pone.0048690.s002.tif]
